# Genotype and phenotype in Parkinson's disease: Lessons in heterogeneity from deep brain stimulation

**DOI:** 10.1002/mds.25535

**Published:** 2013-07-01

**Authors:** Aikaterina Angeli, Niccolo E Mencacci, Raquel Duran, Iciar Aviles-Olmos, Zinovia Kefalopoulou, Joseph Candelario, Sarah Rusbridge, Jennifer Foley, Priyanka Pradhan, Marjan Jahanshahi, Ludvic Zrinzo, Marwan Hariz, Nicholas W Wood, John Hardy, Patricia Limousin, Tom Foltynie, MRCP, MD

**Affiliations:** 1 Sobell Department of Motor Neuroscience, University College London (UCL) Institute of NeurologyQueen Square, London, United Kingdom; 2 Reta Lila Weston Laboratories and Departments of Molecular Neuroscience, UCL Institute of NeurologyQueen Square, London, United Kingdom; 3 Department of Neurology and Laboratory of Neuroscience, “Dino Ferrari” Centre, Università degli Studi di Milano-IRCCS Istituto Auxologico ItalianoMilan, Italy; 4 Department of Neuropsychology, National Hospital for Neurology and NeurosurgeryQueen Square, London, United Kingdom

**Keywords:** genetics, Parkinson's disease, phenotype, deep brain stimulation, heterogeneity

## Abstract

Variation in the genetic risk(s) of developing Parkinson's disease (PD) undoubtedly contributes to the subsequent phenotypic heterogeneity. Although patients with PD who undergo deep brain stimulation (DBS) are a skewed population, they represent a valuable resource for exploring the relationships between heterogeneous phenotypes and PD genetics. In this series, 94 patients who underwent DBS were screened for mutations in the most common genes associated with PD. The consequent genetic subgroups of patients were compared with respect to phenotype, levodopa (l-dopa), and DBS responsiveness. An unprecedented number (29%) of patients tested positive for at least 1 of the currently known PD genes. Patients with Parkin mutations presented at the youngest age but had many years of disease before needing DBS, whereas glucocerebrosidase (GBA) mutation carriers reached the threshold of needing DBS earlier, and developed earlier cognitive impairment after DBS. DBS cohorts include large numbers of gene positive PD patients and can be clinically instructive in the exploration of genotype-phenotype relationships.

Patients with Parkinson's disease (PD) show marked heterogeneity in their clinical features in relation to motor phenotype, rate of progression, and development of cognitive impairment.^1^ Clinical studies of heterogeneity have been complemented by studies of the relation between PD pathophysiological mechanisms and PD risk factors.^2^,^3^ Some aspects of PD heterogeneity almost certainly relate to the recognized genes^5^–^6^ with superimposed environmental, epistatic, as well as stochastic modifiers of PD phenotype. Several studies have described the phenotype of patients with specific genetic mutations^7^,^8^; however, direct comparisons of PD phenotype between different genetically defined groups are scarce.^10^

Patients with PD who undergo deep brain stimulation (DBS) tend to have a young age at onset and, thus, probably include an over-representation of genetic forms of PD. In this study, an extensive genotyping of a series of patients with PD who underwent DBS surgery was performed, and subsequent comparisons were made of clinical phenotypes among genetic subgroups both on and off dopaminergic medication along with responses to DBS.

## Patients and Methods

### Clinical Assessments

All patients were followed at the National Hospital for Neurology and Neurosurgery, Queen Square and underwent DBS between 2002 and 2011. Basic demographic details were recorded. The levodopa (l-dopa) equivalent dose was derived using standard formulae.^11^

The selection of surgical candidates and targets was performed after assessments, including l-dopa challenge, using the Unified Parkinson's Disease Rating Scale (UPDRS) part 3 (motor examination) in the practically defined “OFF-state” and “ON-state” and also using UPDRS parts 1 (nonmotor activities of daily living), 2 (motor activities of daily life), and 4 (motor complications). Additional assessments included brain magnetic resonance imaging and neuropsychological assessments (including the Mattis Dementia Rating Scale [DRS-2]).

UPDRS part 3 subscale scores were derived to quantify tremor (items 20 and 21), akinesia and rigidity (items 22–26 and 31), and axial features (items 18, 19, and 27–30). Part 4 of the UPDRS was divided into items 32 through 34 for dyskinesia and items 35 through 39 to reflect OFF periods.

The optimal DBS target was chosen according to the frequency and severity of OFF-symptoms, dyskinesia, tremor, speech-intelligibility, and cognition. DBS was performed using a standard technique that has been described previously.^12^–^15^ Genotyping consent was obtained from all patients.

Postoperative assessments were performed 12 months after DBS surgery. Patients who refused to have the stimulation switched off had their preoperative “OFF-medication” scores imputed. A subset of 37 patients with >5 years of follow-up post-DBS underwent repeat cognitive assessment and had the mean annual change on the DRS-2 between baseline and follow-up calculated.

### Genotyping

Genetic testing for PD genes was performed in accordance with the service supplied by the Department of Neurogenetics, Queen Square. Multiplex ligation-dependent probe amplification (MLPA) was performed according to manufacturer's instructions using the P051 Salsa MLPA Parkinson probe set (MRC Holland, Amsterdam, the Netherlands). This set includes probes that detect exonic rearrangements in PARK1 (synuclein alpha [SNCA]; exons 1–6), PARK2 (Parkin: exons 1–12), PARK6 (phosphatase and tensin homolog-induced putative kinase 1 [PINK1]; exons 1–8), PARK7 (DJ1; coding exons 3, 5, 6, and 7), PARK8 (leucine-rich repeat kinase 2 [LRRK2]; exons 1, 2, 10, 15, 27, 41, and 49), the Ala30Pro mutation in PARK 1 (SNCA), and the Gly2019Ser mutation in PARK8 (LRRK2). Patients who were identified as positive for G2019S point mutations had their results confirmed by Sanger sequencing.

In addition, the Parkin (12 exons) and glucocerebrosidase (GBA) (11 exons) coding region and the flanking intronic sequences were completely screened by Sanger sequencing. The primers and polymerase chain reaction (PCR) conditions we used are available on request. PCR products were bidirectional sequenced using the BigDye Terminator version 3.1 sequencing chemistry, then loaded on the ABI3730xl genetic analyzer (Applied Biosystems, Foster City, CA).

### Statistical Analysis

All statistical tests were performed using the Stata statistical software package (version 8; StataCorp LC, College Station, TX). The χ^2^ test was used for categorical data. Continuous data were checked for normality, and 1-way analysis of variance was used to compare subgroups. Post hoc comparisons were performed using Sidak's method. Kruskal Wallis and nonparametric post hoc comparisons also were used as appropriate.

## Results

Data are reported for 94 unselected patients with PD who underwent DBS. The mean (± standard deviation [SD]) age of patients at the onset of symptoms was 40.4 ± 8.2 years (range, 7–58 years). Of these 94 patients, 27 (29%) had at least 1 mutation in a PD gene. The results of genotyping for this cohort are presented in Table1.

**Table 1:** Description of abnormal genetic findings in a cohort of 94 patients with PD who underwent deep brain stimulation surgery

Genetic test results	No. of patients (sex)	Description	Mean age ± SD/age at symptom onset, y	Family history^a^
Parkin double mutation carriers			24 ± 11.1	
	4 (3 M, 1 F)	Homozygous [c.101_102delAG]	30	Nil
		c.1289G>A, p.G430D and c.823C>T; p.Arg275Trp	20	Sibling
		c.337_376del and c.465–466del	36	Nil
		Homozygous deletion of exon 3 and 4	27	Sibling
		c.823C>T; p.Arg275Trp and Heterozygous duplication of exon 6	7	Unknown
Parkin single mutation carriers				
	5 (4M, 1F)	c.1000C>T; p.Arg334Cys	41	Nil
		c.337_376del, p.P113TfsX51	39	Nil
		c.1310C>T; p.P437L and GBA T369M^b^	39	Nil
GBA confirmed mutation			42.9 ± 6.2	
	16 (9 M, 7 F)	R463C/R463C	45	Parent
		L444P/E326K	34	Nil
		N370S	45	2 Second-degree relatives
		D409H	39	Nil
		rec*Nci*l	40	Nil
		R463C	45	Parent
		N188S	49	Nil
		R275Q	42	Parent
		IVS2 + 1 G>A	41	Nil
		L444P	45	Nil
		E326K/E326K	42	1 Third-degree relative
		E326K	36	1 Second-degree relative
		E326K	51	Parent
		E326K	58	Nil
		E326K and LRRK2 G2019S^b^	35	Nil
		T369M and parkin c.1310C>T; p.P437L^b^	39	Nil
LRRK2			43 ± 8.7	
	5 (3 M, 2 F)	G2019S	40	Nil
		G2019S	36	Parent
		G2019S	49	Nil
		G2019S	55	Parent
		G2019S and GBA-E326K^b^	35	Nil
No mutation found				
	67 (46 M, 21 F)		40.8 ± 7.2	Parent, n = 9
				Sibling, n = 2
				Grandparent, n = 2
				Half sibling, n = 1
				Cousin, n = 4
				Aunt, n = 2

a Family history data details the number of patients reporting a positive family history of PD, together with affected relative in each genetic subgroup.

b Note that the numbers add up to 96, because 2 individuals who carried 2 confirmed PD mutations are represented twice in the table.

SD, standard deviation; M, males; F, females; GBA, glucosidase beta acid; LRRK2, leucine-rich repeat kinase 2.

### Genotypes

#### Parkin

All single mutation carriers are reported, but we have only included Parkin homozygotes/compound heterozygotes for phenotypic comparisons. Eight individuals had Parkin mutations, of which 5 patients had 2 mutations, and 3 patients had a single mutation (1 of whom also had a GBA T369M mutation).

#### GBA

Sixteen patients had at least 1 mutation in the GBA gene, most common of which were the E326K mutation.^9^–^16^

#### LRRK2-G2019S

Five patients were identified with the G2019S mutation. One patient with a G2019S mutation also carried the E326K GBA mutation. The remaining 67 patients had no mutation detected.

### Phenotypes

The mean age at onset for the individuals who had 2 Parkin mutations (24.0 years) was younger than the age of those who had GBA mutations (*P* = 0.0009) and those without mutations (*P* = 0.006) (Table1).The mean duration of PD (± SD) in patients at DBS surgery was 15.0 ± 6.6 years (range, 3.8–38.0 years). Patients with Parkin mutations had a longer duration of disease at surgery than all other subgroups, whereas patients with GBA mutations had a shorter duration of disease at surgery compared with the Parkin mutation and mutation-negative subgroups (*P* = 0.001) (Table2). l-Dopa challenge revealed a mean UPDRS part 3 improvement of 68% in the group as a whole. UPDRS part 4 dyskinesia scores were higher in the patients with Parkin mutations despite their receipt of lower doses of l-dopa. No other motor phenotypic differences were detectable (Table3).

**Table 2:** Preoperative Unified Parkinson's Disease Rating Scale scores and response to l-dopa according to genetic subgroup

	Mean ± SD										
				UPDRS-II						UPDRS-III									UPDRS-IV										
Genetic test results	Duration of PD at DBS assessment, y	LED, mg	UPDRS-I	Off meds	On meds	Off meds	On meds	Percentage improvement in score with l-dopa	Dyskinesia score	Off meds score
Parkin (compound heterozygotes/homozygotes; N = 5)	25.2 ± 12.8	960 ± 611	1.7 ± 2.1	24.3 ± 4.0	6 ± 6.2	57.0 ± 11.2	21.0 ± 6.4	61.0 ± 18.3	5.3 ± 3.9	3.5 ± 1.3
GBA (confirmed mutation; N = 16)	11.2 ± 5.0	1143 ± 540	1.5 ± 1.5	23.1 ± 12.3	10.9 ± 11.8	51.3 ± 14.0	18.0 ± 15.4	66.9 ± 18.6	3.0 ± 2.7	4.6 ± 2.1
LRRK2 (N = 5)	12.1 ± 1.8	1317 ± 803	1.2 ± 1.1	26.8 ± 7.9	4.5 ± 4.4	65.4 ± 14.9	10.8 ± 5.1	84.9 ± 5.5	4.2 ± 3.1	5.2 ± 1.3
No mutation found (N = 67)	15.1 ± 5.5	1259 ± 559	2.0 ± 1.6	22.9 ± 7.9	8.0 ± 7.2	47.4 ± 14.7	15.6 ± 11.3	68.5 ± 19.3	3.0 ± 2.2	4.5 ± 1.3

SD, standard deviation; UPDRS-I through UPDRS-IV, Unified Parkinson's Disease Rating Scale parts 1 (nonmotor activities of daily living), 2 (motor activities of daily living), 3 (motor examination), and 4 (motor complications), respectively; DBS, deep brain stimulation; meds, medication; LED, l-dopa equivalent dose; GBA, glucosidase beta acid; LRRK2, leucine-rich repeat kinase 2.

**Table:** Preoperative Unified Parkinson's Disease Rating Scale motor scores “off” and “on” l-dopa according to genetic subgroup^a^

	UPDRS score: Mean ± SD						
	Off l-dopa				On l-dopa						
Genetic test results	Tremor	Bradykinesia/rigidity	Axial	Tremor	Bradykinesia/rigidity	Axial
Parkin (compound heterozygotes/homozygotes; N = 5)	6.4 ± 3.8	36.4 ± 7.4	14.2 ± 4.2	1.4 ± 1.7	13.0 ± 4.5	6.6 ± 2.4
GBA (confirmed mutation; N = 16)	6.9 ± 5.1	31.2 ± 11.0	13.1 ± 5.7	1.2 ± 1.9	12.1 ± 11.0	4.7 ± 4.6
LRRK2 (N = 5)	9.4 ± 2.2	40.8 ± 8.8	15.2 ± 6.7	0 ± 0.0	8.3 ± 3.6	2.5 ± 2.1
No mutation found (N = 67)	7.3 ± 5.3	29.4 ± 10.3	10.8 ± 4.8	1.7 ± 3.4	10.3 ± 7.4	3.6 ± 2.9

a There were no significant differences in the motor phenotype according to genetic subgroup. Tremor was seen in all subgroups, and the proportion of tremor compared with akinesia/rigidity or axial features was similar in each group.

UPDRS, Unified Parkinson's Disease Rating Scale; SD, standard deviation; GBA, glucosidase beta acid; LRRK2, leucine-rich repeat kinase 2.

There was a difference in the target choice for DBS according to genotypic subgroups (χ^2^ statistic, 29.1; *P* < 0.001), with an excess of patients who had Parkin and GBA mutations allocated to bilateral globus pallidus internus DBS (GPi-DBS) rather than subthalamic nucleus DBS (STN-DBS). The percentage improvement in the UPDRS part 3 score “OFF-medication” was less with bilateral GPi-DBS than with STN-DBS for all groups (*P* = 0.001) (Table4). Two mutation-negative patients underwent bilateral GPi-DBS and had worse OFF-medication/ON-stimulation scores compared with their preoperative OFF-medication assessment, but both experienced improvement in dyskinesias.

**Table:** Target selection and response to deep brain stimulation according to target and genetic subgroup

				UPDRS-III score: Mean ± SD										UPDRS-IV												
					Post-op										Dyskinesia score: Mean ± SD												
Genetic test results	No. of patients	DBS target	Disease duration at DBS: Mean ± SD, y	Pre-op: Off meds	Off meds/off stim	Off meds/on stim	Percentage improvement in UPDRS-III score off meds/on stim vs pre-op off meds	Post-op: On meds/on stim	Mean ± SD reduction in LED, mg	Pre-op	Post-op	Percentage improvement in post-op vs pre-op UPDRS-IV score
Parkin (compound Hets/Homozy's)	3	GPi	21.1 ± 16.2	53.3 ± 13.9	43.3 ± 16.4	42.0 ± 19.0	21%	27.3 ± 17.6	−237 ± 315	8.0 ± 1.4	1.67 ± 2.0	70%
	2	STN	31.3 ± 0.6	62.5 ± 3.5	84.0 ± 22.6	43.0 ± 0.0	31%	23.5 ± 6.4	20 ± 594	2.5 ± 3.5	2.0 ± 1.4	20%
GBA (confirmed PD mutation)	2	GPi	15.5 ± 5.3	64.5 ± 21.9	66.5 ± 19.1	50.0 ± 19.8	22%	41.0 ± 15.6	1005 ± 77	8.5 ± 0.7	0.5 ± 0.7	94%
	13	STN	10.9 ± 4.9	50.5 ± 12.4	56.1 ± 18.8	28 ± 11.4	40%	15.9 ± 10.4	146 ± 510	2.4 ± 1.7	1.5 ± 1.6	37%
	1	VIM (unilateral)	3.9	35	35	20	43%	8	445	0	0	—
LRRK2	5	STN	12.1 ± 1.8	65.4 ± 14.9	69.2 ± 12.4	30.6 ± 16.1	53%	14.0 ± 8.1	586 ± 495	4.2 ± 3.1	1.4 ± 2.6	50%
No mutation found	2	GPi	21.7 ± 0.5	40.5 ± 13.4	78 ± 7.1	51.0 ± 7.1	−28%	25.5 ± 4.9	227 ± 89	6.0 ± 1.4	3.5 ± 0.7	42%
	65	STN	14.8 ± 5.4	47.6 ± 14.8	50.0 ± 15.4	24.6 ± 11.3	48%	15.0 ± 9.0	468 ± 494	3.1 ± 2.2	3.0 ± 2.2	26%

SD, standard deviation; UPDRS-III, Unified Parkinson's Disease Rating Scale, part 3 (motor examination); meds, medication; stim, stimulation; LED, l-dopa equivalent dose; UPDRS-IV, Unified Parkinson's Disease Rating Scale, part 4 (motor complications); DBS, deep brain stimulation; GPi, globus pallidus internus; STN, subthalamic nucleus; GBA, glucosidase beta acid; VIM, ventral intermediate nucleus; LRRK2, leucine-rich repeat kinase 2.

There was no significant difference in the degree of improvement achievable with STN-DBS between mutation-negative patients and patients who had Parkin, GBA, or LRRK2 mutations. There was a worsening of postoperative OFF-medication/OFF-stimulation scores compared with preoperative OFF-medication scores in patients who underwent STN-DBS. Note that medication doses are substantially reduced postoperatively; therefore, the practically defined “OFF” may be closer to the true “OFF” at this time point.

Longitudinal 5-year follow-up data with respect to cognition were available for 35 individuals; all had undergone STN-DBS, and 6 had GBA mutations. The mean ± SD decline in Mattis DRS-2 scores for patients with GBA mutations was 4.4 ± 7.3 points per year compared with 0.5 ± 0.9 points per year among mutation-negative patients.

## Discussion

The results from this study confirm that patients who undergo DBS surgery are a valuable resource for identifying genetic forms of PD. The frequency of mutation-positive patients with PD was much greater in our cohort (27 of 94 patients; 29%) than in population-representative cohorts of PD.^17^ Although the use of DBS cohorts facilitates the rapid identification of mutation-positive patients for study, patients who have major cognitive or psychiatric problems or a relative lack of l-dopa response are excluded; therefore, the relevance of such data to the broader population of PD patients is partially limited. Patients in our cohort underwent detailed screening for the most common PD genes; nevertheless, not every gene previously linked to PD was sequenced. Therefore, some patients with genetic forms of PD may have been misclassified as mutation-negative.

Apart from confirming that patients with Parkin mutations had more severe l-dopa–induced dyskinesia, no consistent phenotypic difference was identified between mutation-positive and mutation-negative subtypes *at a single time point*. This said, the small numbers of patients in each genetic subgroup, together with variable disease duration between groups, limits the power of this study to reach definite conclusions. In a previous study that examined the phenotype of GBA patients, bradykinesia as a presenting feature was the only difference compared with GBA-negative patients.^9^ Together with our data, this suggests that the currently known genetic mutations all lead to a similar pattern/variety of patterns of neurodegeneration.

However, longitudinal data are more revealing. Disease duration at DBS differs significantly between genetic subgroups. Patients with Parkin mutations are younger at the onset of disease and have earlier dyskinesia, but they have longer disease duration before DBS, indicating a more indolent form of the disease. The patients in our study who had Parkin mutations were receiving lower doses of l-dopa replacement than all other groups, probably because of their increased severity of dyskinesia. PD patients with GBA mutations required DBS earlier in their disease course, because they developed disabling motor symptoms at an earlier stage despite conventional oral treatment, consistent with other published studies suggesting that patients with PD who have GBA mutations also have higher rates of cognitive decline (irrespective of DBS).^18^

Previous reports have documented the generally good response to DBS of small numbers of patients with Parkin,^19^–^20^ Pink-1,^19^–^20^ and LRRK-2 mutations.^19^,^21^ However, the long-term response of patients with GBA mutations to DBS is less well known. In the current series, the response to either STN-DBS or GPi-DBS did not differ significantly between the subgroup with GBA mutations (at 1 year) and any other subgroup. However, our longitudinal follow-up of the subgroup with GBA mutations suggested a faster rate of cognitive decline after DBS. A previous report of 3 patients with GBA mutations who underwent STN-DBS also identified a more aggressive process that led to both cognitive impairment and axial impairments^23^; whereas, of 2 other patients with GBA mutations who underwent STN-DBS, 1 had early cognitive decline, and the second had persistent benefit.^9^ Whether there is an interaction between GBA status and the risk of subsequent cognitive decline after STN-DBS needs to be clarified.

More aggressive disease in patients who have GBA mutations may have relevance from a therapeutic standpoint in terms of supporting the earlier introduction of l-dopa and possibly the use of bilateral GPi-DBS rather than bilateral STN-DBS (because there are likely to be additional negative cognitive consequences of STN-DBS^24^). Further prospective data would be needed to confirm this. Our data support the idea that the heterogeneity of PD should be considered longitudinally in terms of the rate of progression of motor and nonmotor features rather than the detailed scrutiny of purely cross-sectional data.

## Author Roles

1. Research Project: A. Conception, B. Organization, C. Execution; 2. Statistical Analysis: A. Design, B. Execution, C. Review and Critique; 3. Manuscript Preparation: A. Writing the First Draft, B. Review and Critique.

A.A.: 1C, 3A

N.E.M.: 1C

R.D:1C

I.A.-O.: 1C

Z.K.: 1C

J.C.: 1C

S.R.: 1C

J.F.: 1C

P.P.: 1C

M.J.: 1C

L.Z.: 1C

M.H.: 1C

N.W.: 1C

JH: 1B, 1C

P.L.: 1B, 1C

T.F.: 1A, 1B, 1C, 2A, 2B, 3B

## Financial Disclosures

This work was undertaken at UCL/UCLH and was partly funded by the Department of Health NIHR Biomedical Research Centres funding scheme. The Unit of Functional Neurosurgery, Queen Square, London is supported by the Parkinson's Appeal for Deep Brain Stimulation, the Edmond J. Safra Philanthropic Foundation, and the Monument Trust. Dr. Foltynie has received honoraria for speaking at meetings sponsored by St. Jude Medical, Genus Pharmaceuticals, and Abvie Pharmaceuticals. He is on the Oxford Biomedica Advisory Board and receives grant support from Cure Parkinson's Trust, Parkinson's UK, Brain Research Trust, and the European Union FP-7 funding scheme. Raquel Duran was funded by a postdoctoral fellowship from the Alfonso Martin Escudero Foundation (Spain).
